# Multi-Scale Spatial Attention-Based Multi-Channel 2D Convolutional Network for Soil Property Prediction

**DOI:** 10.3390/s24144728

**Published:** 2024-07-21

**Authors:** Guolun Feng, Zhiyong Li, Junbo Zhang, Mantao Wang

**Affiliations:** College of Information Engineering, Sichuan Agricultural University, Ya’an 625014, China; fengguolun@stu.sicau.edu.cn (G.F.); lzy@sicau.edu.cn (Z.L.); 2021219004@stu.sicau.edu.cn (J.Z.)

**Keywords:** soil, vis-NIR spectroscopy, convolutional neural networks, spatial attention mechanism

## Abstract

Visible near-infrared spectroscopy (VNIR) is extensively researched for obtaining soil property information due to its rapid, cost-effective, and environmentally friendly advantages. Despite its widespread application and significant achievements in soil property analysis, current soil prediction models continue to suffer from low accuracy. To address this issue, we propose a convolutional neural network model that can achieve high-precision soil property prediction by creating 2D multi-channel inputs and applying a multi-scale spatial attention mechanism. Initially, we explored two-dimensional multi-channel inputs for seven soil properties in the public LUCAS spectral dataset using the Gramian Angular Field (GAF) method and various preprocessing techniques. Subsequently, we developed a convolutional neural network model with a multi-scale spatial attention mechanism to improve the network’s extraction of relevant spatial contextual information. Our proposed model showed superior performance in a statistical comparison with current state-of-the-art techniques. The RMSE (R²) values for various soil properties were as follows: organic carbon content (OC) of 19.083 (0.955), calcium carbonate content (CaCO_3_) of 24.901 (0.961), nitrogen content (N) of 0.969 (0.933), cation exchange capacity (CEC) of 6.52 (0.803), pH in H_2_O of 0.366 (0.927), clay content of 4.845 (0.86), and sand content of 12.069 (0.789). Our proposed model can effectively extract features from visible near-infrared spectroscopy data, contributing to the precise detection of soil properties.

## 1. Introduction

Soil is a critical natural resource, and the accurate and timely acquisition of soil property information is essential for ensuring soil health and achieving sustainable agriculture [1]. Traditional methods typically entail on-site sampling and laboratory testing; however, these approaches are plagued by high costs, low efficiency, and environmental unfriendliness. In recent years, soil visible–near-infrared reflectance spectroscopy has emerged as a rapid, cost-effective, environmentally friendly, non-destructive, and reproducible analytical technique [2]. Therefore, it has gradually emerged as an effective alternative to traditional methods. However, soil property prediction is challenging due to the spectral data’s numerous spectral bands, strong collinearity, and intricate interrelationships. With the advancement of machine learning, numerous nonlinear regression algorithms have been developed and applied. Said et al. [3] conducted a comparative analysis of three regression techniques—Partial Least Squares Regression (PLSR), Support Vector Machine (SVM), and Multivariate Adaptive Regression Splines (MARS)—for the prediction of the organic matter and clay content in saline soils. Similarly, Yang et al. [4] employed four methods—PLSR, Least Squares Support Vector Machine (LS-SVM), Extreme Learning Machine (ELM), and the Cubist regression model—to forecast the soil organic matter and pH levels. Notwithstanding these advancements, these machine learning methods demonstrate computational efficiency and modeling capability limitations.

In contrast to conventional machine learning methods, deep learning models, particularly convolutional neural networks (CNNs), are highly effective in multi-dimensional data and large-scale problems due to their hierarchical structure, and the learning capabilities of the patterns of complex problems [5]. They have been extensively utilized across domains such as image classification [6,7], natural language processing [8], and speech recognition [9]. By leveraging sparse local connections and weight sharing, CNNs have been proven to effectively and automatically learn and extract local and abstract features from complex spectral data [10]. By stacking multiple convolutional and pooling layers, CNNs can efficiently capture intricate patterns within the data, making them well-suited for soil property prediction tasks [11]. In recent years, the application of deep learning in soil spectroscopy has become increasingly widespread. In 2015, Veres et al. [12] pioneered the integration of deep learning into soil spectroscopy, successfully validating the efficacy of one-dimensional convolutional neural networks (1D CNNs) in predicting specific soil properties. To extract deep feature information, Zhong et al. [13] proposed deep CNN models for the regression prediction of seven soil properties. Spectral data are commonly considered to exhibit a temporal structure [14]. The presence of identical feature peaks at different positions in spectral data may indicate varying information, and the sequential nature of spectral data can affect the accuracy of soil property predictions [15]. However, convolutional neural networks (CNNs) are insensitive to positional information during data extraction, which can lead to a decline in model performance. To address this issue, some studies have adopted recurrent neural networks (RNNs), which are better suited for handling sequential data. RNNs can use feedback connections to store historical information over time. Singh et al. [16] used long short-term memory (LSTM) to predict six soil physical and chemical properties from the LUCAS spectral library. The network can effectively capture and retain short-term and long-term dependencies in sequential data. Yang et al. [17] proposed a novel approach, the Combined CNN and RNN model (CCNVR), that exploits the strengths of both Convolutional Neural Networks (CNNs) and Recurrent Neural Networks (RNNs). Initially, the model employs CNN to extract features from the raw soil spectra. Subsequently, it utilizes a RNN to analyze the relationships among these features. This integration method effectively distills soil spectral features while also profoundly investigating the interconnections among these features. Furthermore, certain studies use two-dimensional transformations to convert one-dimensional spectral data into two-dimensional spectral images to enhance the feature extraction capabilities of the model. Padarian et al. [18] employed a short-time fast Fourier transformation to convert the raw spectra from the LUCAS database into two-dimensional spectrograms. Then, they used a 2D multi-task CNN to predict six soil properties. Li et al. [19] similarly used a short-time fast Fourier transformation to construct a dual-stream convolutional neural network model (Multi-CNN), which integrates both one-dimensional and two-dimensional convolutions to achieve accurate the prediction of multiple soil properties. Jin et al. [20] investigated four methods for converting one-dimensional spectra into two-dimensional spectral images: slicing and reshaping, the Gramian angular difference field, the Gramian angular field, and the Markov transition field. They combined the transformed images with the Swin Transformer to predict six soil properties. Additionally, they demonstrated that the spatial positional correlations preserved in the Gramian angular field method could enhance the information extraction capability of deep neural networks.

This paper introduces a multi-scale spatial attention mechanism module to tackle the issues previously outlined. The spatial attention mechanism, a pivotal element within convolutional neural networks, functions as an adaptive process that selectively focuses on key spatial areas, thus addressing the question of “where to focus” [21]. This approach significantly improves the network’s capacity to discern essential objects within the feature maps by identifying and emphasizing critical regions. It accomplishes this through the application of weighted operations across different areas of the input feature map along the spatial dimension, allowing the network to give precedence to pertinent information [22,23]. We aim to enhance the prediction of soil properties by employing a multi-scale spatial attention mechanism. This mechanism captures information at different scales using convolutional kernels of varying sizes, thereby improving the feature extraction capabilities of convolutional neural networks.

Furthermore, researchers utilize various algorithms to preprocess spectral data to advance the creation of more robust calibration models for soil property prediction. This preprocessing endeavor aims to diminish or eradicate noise in the spectra while highlighting relevant information. Ultimately, this assists calibration models in recognizing the correlation between the input spectra and output soil properties [24]. Common soil spectral preprocessing methods include Savitzky–Golay smoothing, standardization, and normalization techniques. Zhao et al. [25] employed four preprocessing methods—first-order derivative, standard normal variate transformation, multiple scatter correction, and detrending—to process the original spectra. Tsakiridis et al. [26] utilized absorbance spectra and some preprocessed spectra developed using standard techniques as one-dimensional multi-channel inputs for their model. It has been confirmed that effectively combining different preprocessing techniques in one-dimensional multi-input methods produces more robust prediction results than single-input methods. However, research on two-dimensional multi-channel inputs in soil visible–near-infrared spectroscopy prediction studies is scarce. We aim to explore whether two-dimensional multi-channel input methods can improve the prediction accuracy, thus providing more reliable tools for soil property analysis.

## 2. Materials and Methods

### 2.1. The Soil Dataset

The soil spectral dataset utilized in this study is derived from the LUCAS soil spectral dataset. This dataset, collected during the 2009 survey, includes 19,036 topsoil samples from 23 European Union countries. All samples underwent standardization and chemical analysis to determine their primary topsoil characteristics, such as coarse fragments, particle size distribution (clay, silt, and sand), pH, organic carbon, carbonates, soluble phosphorus, total nitrogen, extractable potassium, and cation exchange capacity. Spectral data were captured using a diffuse reflectance spectrometer (XDS™ Rapid Content Analyzer, NIRSystems, Inc., Laurel, MD, USA) across a range of 400–2500 nm with a spectral resolution of 0.5 nm, resulting in 4200 data points per sample [27,28,29]. In this study, seven soil properties were selected as target prediction variables: the calcium carbonate content (CaCO_3_, g·kg^−1^), cation exchange capacity (CEC, cmol(+)·kg^−1^), clay fraction (Clay, %), sand fraction (Sand, %), nitrogen content (N, g·kg^−1^), organic carbon content (OC, g·kg^−1^), and pH in H_2_O (pH). We considered all available soil samples in the dataset, encompassing both mineral and organic soils, without considering any additional information such as geographic origin or soil category.

### 2.2. Method

The entire experimental process was divided into three parts. First, the raw data underwent various preprocessing techniques. Second, the one-dimensional data were transformed into two-dimensional spectral images using the Gramian Angular Difference Field transformation. Next, the best combination of preprocessing methods for different soil properties for a multi-channel input was analyzed using the Vgg16 network model [30]. Finally, the proposed deep learning model was employed to achieve high-precision predictions of soil property.

#### 2.2.1. Preprocessing Methods

**Figure 1:**
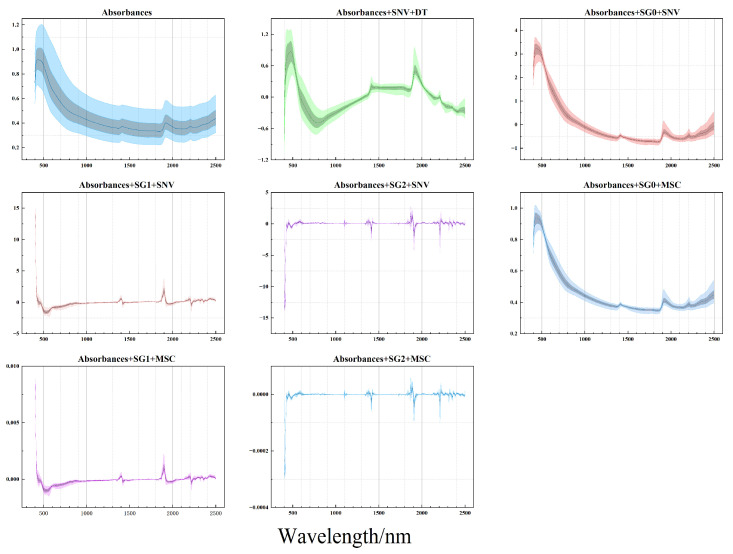
The initial absorbance spectra and the seven corresponding spectral preprocessing methods. The 5th, 16th, 50th, 84th, and 95th percentiles are depicted.

Spectral preprocessing techniques optimize raw spectral data, providing more accurate inputs for subsequent analysis and modeling and also acquiring various spectral information through different preprocessing methods that complement each other. To fully leverage this complementary information, we selected spectra processed with a series of common preprocessing methods, along with the original absorbance spectra, as multi-channel inputs for the model, with each spectrum forming an independent channel. Several preprocessing methods commonly used in soil science (such as SG filtering, standard normal variate transformation, and scatter correction) were chosen to create a spectral information pool. The following seven methods were selected to transform the original absorbance spectra: (1) standard normal variate transformation followed by detrending (SNV + DT); (2) the zero-order Savitzky–Golay filter with a window width of 9, followed by standard normal variate transformation (SG0-SNV); (3) the first-order Savitzky–Golay filter with a window width of 9, followed by standard normal variate transformation (SG1-SNV); (4) the second-order Savitzky–Golay filter with a window width of 9, followed by standard normal variate transformation (SG2-SNV); (5) the zero-order Savitzky–Golay filter with a window width of 9, followed by multiple scatter correction (SG0-MSC); (6) the first-order Savitzky–Golay filter with a window width of 9, followed by multiple scatter correction (SG1-MSC); and (7) the second-order Savitzky–Golay filter with a window width of 9, followed by multiple scatter correction (SG2-MSC). The original spectra and the corresponding spectral transformations are depicted in Figure 1.

#### 2.2.2. 2D Transformation Methods

In time series processing, the Gramian Angular Field (GAF) method [31] transforms time series data into image data. This technique retains the complete information of the signal while preserving its temporal dependencies. Visible near-infrared spectroscopy can be viewed as a type of time series. Utilizing the GAF transformation to preserve the spatial position correlations of spectral sequences enables data augmentation and improves the information extraction ability of neural networks [20]. After converting sequence data into image data, we can fully utilize the advantages of convolutional neural networks in image classification and recognition and explore the methods suitable for deep learning algorithm models. We can obtain a two-dimensional GAF image for a given sequence $X=\{x_{t},1,2,\ldots ,M\}$ by following the steps listed below: To reduce the dimensionality of the sequence, this study employs the Piecewise Aggregate Approximation (PAA) method [32]. Using this method, we obtain the aggregated sequence $\bar{X}=\left\{{\bar{x}}_{t},t=1,2,\ldots N\right\}$. It should be noted that in this study, the value of N is set to 64. The formula for the sequence $\bar{X}$ is as follows:

(1) $$\bar{X_{t}}=\frac{1}{k}\sum_{j=k*(t-1)+1}^{k*t}x_{t},1\leq t\leq M,$$

where $k=\frac{M}{N},N<M$;

Next, the data obtained from the first step $\bar{X}$ need to be processed using min–max normalization to scale its range to [0, 1]. This will result in a new data set $\tilde{\bar{X}}$. The specific transformation method is shown in Equation (2).

(2) $$\tilde{\bar{x_{t}}}=\frac{\left(\bar{x_{t}}-{\bar{x_{t}}}_{\min}\right)}{{\bar{x_{t}}}_{\max}-{\bar{x_{t}}}_{\min}}$$

For the data obtained in the second step $\tilde{\bar{X}}$, a polar coordinate transformation can be applied to obtain the corresponding angle and radius for each data point.

(3) $$\left\{\begin{array}{l} \phi_{i}=\arccos(\tilde{\bar{x_{t}}}),-1\leq \tilde{\bar{x_{t}}}\leq 1,\tilde{\bar{x_{t}}}\in \tilde{\bar{X}}\\ r=\frac{t}{N},t\in N \end{array}\right.,$$

where $\;\phi_{i}$ is the angle and r is the radius;

Using Equations (4) and (5), the cosine of the sum of the angles and the sine of the difference between the angles for two different points can be calculated. Consequently, the Gramian Angular Summation Field ($X_{GASF}$) and Gramian Angular Difference Field ($X_{GADF}$) can be obtained.

(4) $$X_{GASF}=\left[\cos(\phi_{i}+\phi_{j})\right]$$

(5) $$X_{GADF}=\left[\sin(\phi_{i}-\phi_{j})\right]$$

**Figure 2:**
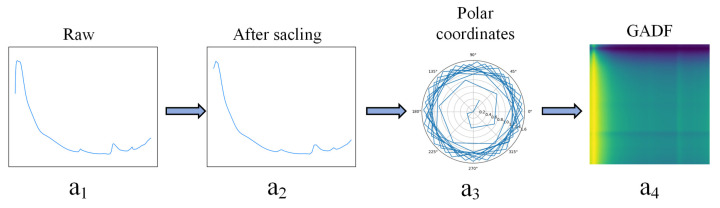
The procedure for converting a visible–near-infrared spectral sequence into a GADF image is as follows: (**a1**) is the original spectral sequence, (**a2**) is the spectral sequence after PAA dimensionality reduction, (**a3**) is the polar coordinate transformation, and (**a4**) is the resulting GADF image.

In this study, we applied the GADF transformation, as shown in Figure 2.

#### 2.2.3. Construction of Multi-Channel Input

To validate the effectiveness of the GADF method, we generated single-channel 2D images from the original soil spectral data. The original spectral sequences and the 2D images were used to train 1D_Vgg16 and 2D_Vgg16 models. Table 1 presents the 2D_Vgg16 network framework in detail. The following hyperparameters were used: SGD was the optimizer, the learning rate was 0.001, the mean squared error was the loss function (MSELoss), the training batch size was 64 samples, and there were 100 training epochs. With the network structure and hyperparameters fixed, only the input data could affect the prediction results.

Next, we applied the preprocessing methods mentioned in Section 2.2.1 to the original spectral sequences, obtaining a series of spectral information. Subsequently, we transformed the spectral information into 2D images. We combined these image data in various ways to construct input data with different channel numbers, which were then fed into the 2D_VGG16 model for training.

To investigate the relationship between the soil property prediction performance and the number of channels in the preprocessing method combination, we gradually increased the number of considered channels to observe the variations in the prediction performance of different properties. Firstly, considering only one channel, we selected one of the preprocessing methods mentioned earlier and obtained a one-channel spectral image by using a two-dimensional transformation as the input variable, denoted as NCC_1_. Next, considering two channels, we selected any two preprocessing methods and obtained a two-channel spectral image by using a two-dimensional transformation as the input variable, denoted as NCC_2_, and so on for other channels. According to the permutation and combination methods, the number of NCC_1_ and NCC_2_ combinations was 8 and 28, respectively (Table 2). Finally, we compared the prediction accuracy of each property under different channel inputs. We selected the preprocessing method combination with the highest prediction accuracy for each property as the input for that property’s multi-channel, two-dimensional image.

#### 2.2.4. Structure of the CNN Network

**Figure 3:**
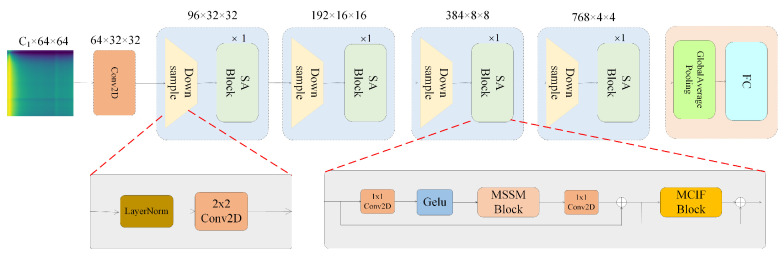
The overall framework of the CNNSANet.

As illustrated in Figure 3, this paper introduces a two-dimensional convolutional neural network model with a spatial attention mechanism called CNNSANet. The model employs a hierarchical architecture divided into four stages, akin to certain studies in computer vision [33,34,35]. Each stage comprises a downsampling layer followed by a sequential stack of blocks. Each block contains a multi-scale spatial selection mechanism module and a multi-channel information fusion module. Downsampling is performed using layer normalization and a 2 × 2 convolution layer with a stride of 2.

**Figure 4:**
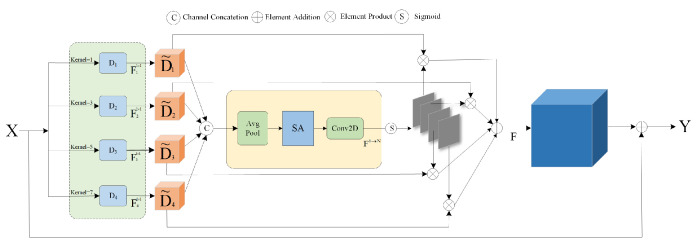
Multi-scale spatial selection mechanism model.

To enhance the network’s focus on the most relevant spatial contextual information, we introduce a Multi-Scale Spatial Selection Mechanism (MSSM), as illustrated in Figure 4. This module can select feature maps from convolutional kernels at different scales. First, to extract rich contextual information features from the input X, we utilize a series of depth-wise separable convolutions with varying receptive fields.

(6) $$D_{0}=X,D_{i}=F_{i}^{dw}(D_{0})$$

Here, $F_{i}^{dw}(•)$ represents a depthwise separable convolution with a kernel size of *k_i_*. Assuming there are *N* convolutional kernels, each kernel is further refined by a 1 × 1 convolution $F_{i}^{dw}(•)$, as shown in Equation (7).

(7) $$\tilde{D_{i}}=F_{i}^{1\times 1}(D_{i}),\;\mathrm{for}\;\;i\;\;\mathrm{in}\;\;[1,N]$$

To obtain more detailed and comprehensive feature information, it is possible to concatenate features obtained from different convolutional kernels with varying receptive field sizes. This approach offers the advantage of fully leveraging the multi-level information extraction capabilities of different convolutional kernels on the image, thereby further enhancing the model’s representative capacity and performance.

(8) $$\tilde{D}=[{\tilde{D}}_{1}\ldots {\tilde{D}}_{N}]$$

Next, we employ the channel-wise average pooling method (represented as $P_{avg}(•)$) to process the spatial features, resulting in the spatial feature map *SA* being obtained through average pooling. Then, through convolutional processing, we transform the pooled features (with only one channel) into *N* spatial attention maps, denoted as $\tilde{SA}$.

(9) $$SA=P_{avg}(\tilde{D})$$

(10) $$\tilde{SA}=F^{1\to N}(SA)$$

To acquire individual spatial selection masks for each convolutional kernel, we apply the Sigmoid activation function to process each spatial attention map ${\tilde{SA}}_{i}$

(11) $${\tilde{SA}}_{i}=\sigma ({\tilde{SA}}_{i})$$

Here, σ(•) denotes the Sigmoid function. Following this, a corresponding spatial selection mask is employed to apply weights to the features extracted by various convolutional kernels. The weighted features are then combined using a convolutional layer F(•), thereby producing the attention feature *S*:

(12) $$S=F(\sum_{i=1}^{N}{\tilde{SA}}_{i}\cdot {\tilde{D}}_{i})$$

Finally, the input feature *X* is multiplied elementwise with *S*, yielding the final output *Y*.

(13) Y=X⋅S

**Figure 5:**
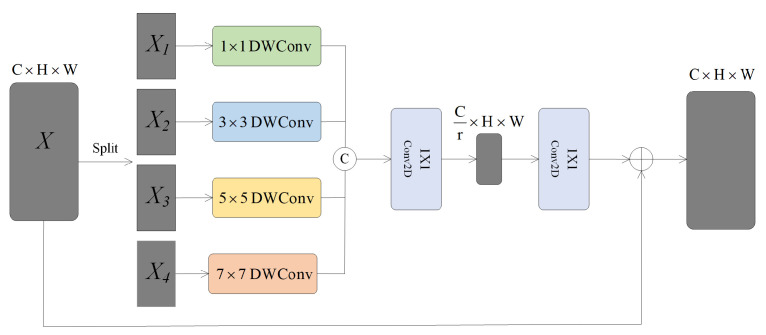
Multi-scale channel information fusion model.

Furthermore, we propose a Multi-Scale Channel Information Fusion (MCIF) module to enhance the model’s representative ability and performance, as depicted in Figure 5. This module improves the network’s ability to learn complex features and enhance information fusion between channels. The MCIF module consists of the following components: a parallel depthwise convolution module with four different scales, a 1 × 1 convolution for channel compression and expansion to reduce the computational cost, and a residual connection. In the parallel depthwise convolution module with four different scales, each convolution processes one-fourth of the channels. The depthwise convolution kernels with sizes {3, 5, 7} effectively capture multi-scale information. The 1 × 1 depthwise convolution kernel also acts as a learnable channel-wise scaling factor, further enhancing the module’s performance. This design ensures that features at different scales are fully utilized, improving the model’s ability to recognize and learn complex features. Furthermore, the 1 × 1 convolution for channel compression and expansion helps reduce the computational costs. Finally, the residual connection better preserves and transmits the information about the original features. The following equation can represent the MCIF module:

(14) $$\mathrm{MCIF}(X)=Conv_{1\times 1}^{\frac{C}{r}\to C}(Conv_{1\times 1}^{C\to \frac{C}{r}}(\sum_{i}^{N}concat(DWConv_{k\times k}(X_{i}))))+X,k=2i-1,N=1,2,3,4$$

(15) $$X_{1},X_{2},X_{3},X_{4}=torch.chunk(X,4,dim=1)$$

### 2.3. Evaluation

The Root Mean Square Error (RMSE), Coefficient of Determination (R^2^), and Ratio of Performance to Inter-Quartile Distance (RPIQ) are utilized to assess the training model’s performance. These metrics are validated on the test set, facilitating an objective and thorough evaluation of the model’s performance. RMSE is used to quantify the discrepancy between the predicted values and the actual observations, and it is calculated as follows:

(16) $$RMSE=\sqrt{\frac{1}{n}\sum_{i=1}^{n}{\left(y_{i}-{\hat{y}}_{i}\right)}^{2}}$$

R^2^ is a statistical indicator used to evaluate the fit of a regression model. It represents how the model explains the variance in the actual data. The R^2^ values range between 0 and 1, with higher values signifying the greater explanatory capability of the model. The calculation formula for R^2^ is as follows:

(17) $$R^{2}=1-\frac{\sum_{i=1}^{n}{\left(y_{i}-{\hat{y}}_{i}\right)}^{2}}{\sum_{i=1}^{n}{\left(y_{i}-{\bar{y}}_{i}\right)}^{2}}$$

The RPIQ is used to measure the deviation between the predicted values and observed values. IQR represents the interquartile range of the observed values, while RMSE is the root mean square error between the predicted and observed values. The formula for calculating the RPIQ is as follows:

(18) $$RPIQ=\frac{IQR}{RMSE}$$

All deep learning models were trained and tested on a single machine. They were implemented using PyTorch (version 1.11.0), and the training process was accelerated with an NVIDIA TITAN V 12GB GPU.

## 3. Results and Discussion

Before the experiment, we randomly split the spectral dataset into two subsets, with 70% of the data used for training and 30% for independent testing. The descriptive statistics for the seven soil properties of the calibration and test set samples are summarized in Table 3. The soil properties show a wide range of values, and the means and standard deviations of the soil properties in the calibration and test sets are similar, indicating a uniform distribution, indicating that the dataset was divided reasonably. We split the training set into five subsets using a five-fold cross-validation method for improving the model’s generalization performance. Specifically, the training dataset was randomly divided into five equal-sized subsets. Then, we performed five iterations of training and validation. In each iteration, one subset was used as the validation set, while the remaining four subsets were used as the training set. Each iteration yielded a model, which we evaluated on the independent test set. The final evaluation result of the model was obtained by averaging the performance metrics of the five models generated from the five iterations.

### 3.1. Analysis of 2D Multi-Channel Inputs

**Figure 6:**
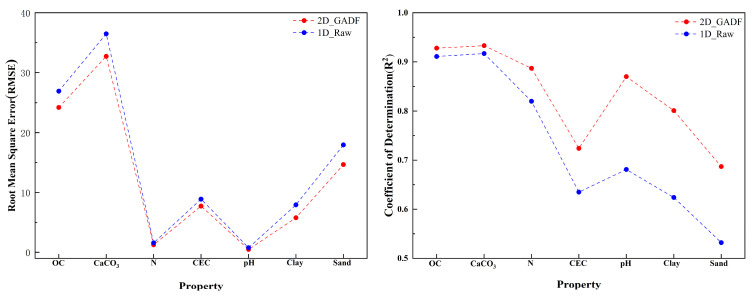
RMSE and R^2^ comparison between 1D raw spectral data and 2D single-channel GADF images constructed using the same 1D raw spectral data as inputs.

Initially, we verified the effectiveness of the GADF method. As seen in Figure 6, the test performance of converting original spectral information into single-channel GADF images outperformed that of the 1D spectral sequences for each soil property. This observation indicates that preserving spatial positional correlations in the GADF method can enhance the information extraction capability of convolutional neural networks.

**Figure 7:**
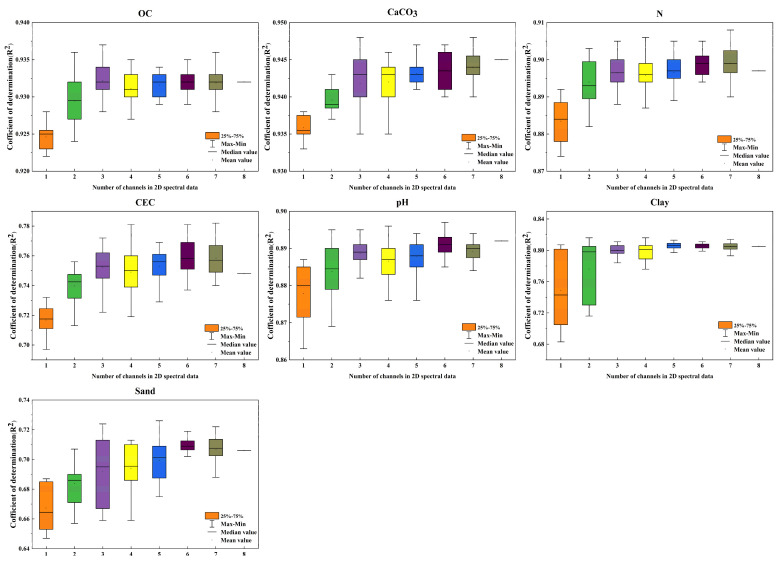
Boxplot of prediction accuracies for different properties of 2D inputs constructed from spectral information obtained using various preprocessing methods and raw spectral information.

Table 4 shows the prediction accuracy for various soil properties using single-channel inputs built from the spectral information obtained via the proposed preprocessing methods and raw spectral information. For different soil properties, the improvement in model performance using different preprocessing combinations is limited, with some combinations even causing a decline in performance. For the five soil properties of CaCO3, N, CEC, pH, and Clay, the preprocessing methods that yielded the best prediction performance for single-channel 2D inputs were SG0 + SNV, SG1 + SNC, SG2 + SNV, SG0 + MSC, and SNV + Detrend, respectively. Compared to the results without using any preprocessing methods, the R^2^ increased by 0.5−1.1%, while the RMSE values decreased by 1.3−5.9%. However, for the soil properties of OC and Sand, applying the previously mentioned preprocessing methods resulted in a decrease in model performance. This suggests that the single-channel 2D inputs created using these preprocessing techniques do not effectively enhance the relative positional information, leading to limited improvements in the prediction accuracy of the soil property content. Figure 7 illustrates the box plots representing the prediction accuracy for different soil properties using spectral information derived from various preprocessing methods and the original spectral data used to form different multi-channel 2D inputs. The outcomes are primarily consistent across different soil properties. Compared to the prediction accuracy of single-channel 2D inputs, the average coefficient of determination for multi-channel 2D inputs demonstrates a marked improvement and a significant reduction in RMSE. For instance, for OC, the RMSE of its multi-channel 2D input decreased by 3.06−6.51%, and the R^2^ increased by 0.4−1.0%. However, the prediction accuracy for different soil properties does not always positively correlate with the number of channels. By comparing the average R² of different multi-channel inputs, the optimal number of channels for each property can be determined, and the combination of preprocessing methods that yield the highest R² for that multi-channel input can then be selected. For OC, the optimal number of channels is three, with the highest prediction accuracy achieved using a three-channel 2D input constructed with SNV, SG1 + MSC, and SG2 + MSC methods. The optimal number of channels is seven for CaCO3, N, and CEC, eight for pH, five for Clay, and six for Sand. Table 5 presents the optimal number of channels for each property, the highest accuracy corresponding to that number of channels, and the preprocessing methods used. These findings suggest that multi-channel two-dimensional images constructed with diverse preprocessing methods can enrich the input information, facilitate data augmentation, and improve the predictive performance of soil properties.

### 3.2. Training and Evaluating the CNNSANet Model

**Figure 8:**
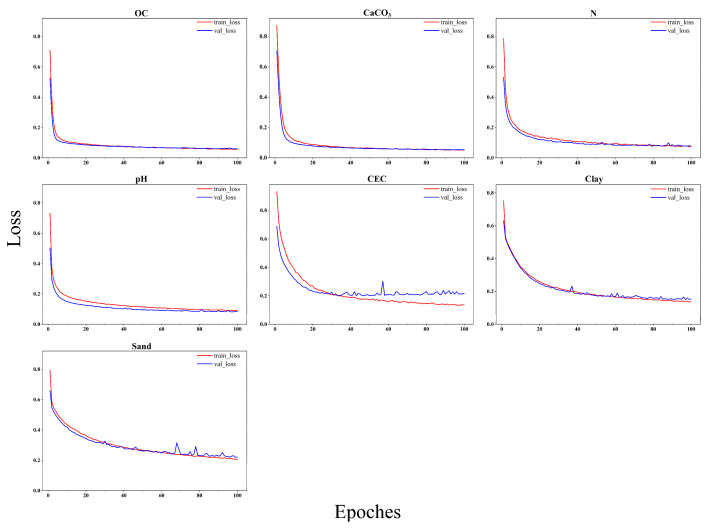
Training and validation losses of the CNNSANet model for seven soil properties.

Based on the multi-channel input analysis experiment results, we selected the 2D spectral images with the optimal number of channels for different properties as inputs (Table 5). Subsequently, we used the proposed CNNSANet model to predict seven soil properties. In our experiment, the loss function was the root mean square error, and we used stochastic gradient descent (SGD) with a batch size of 64. Figure 8 shows the loss variation over 100 training iterations. For the prediction tasks of the seven soil properties, the training loss and validation loss for OC, CaCO_3_, N, pH, and Clay decreased rapidly during the first 0−10 epochs and then stabilized, with the training and validation loss curves almost overlapping. For the soil properties CEC and Sand, the training loss and validation loss decreased slowly, and the validation loss exhibited significant fluctuations. This indicates that the prediction performance for these two properties is not as strong as for the other five properties. Overall, the loss of each model decreases with increasing training iterations, indicating that our models perform well in predicting soil properties and exhibit strong generalization capabilities. To evaluate the effectiveness of the MSSM block and MCIF block in the CNNSANet model, we conducted ablation experiments on our proposed spatial attention mechanism module as follows: We used single-channel 2D images constructed from raw spectra and multi-channel 2D images constructed using different optimal preprocessing methods for each soil property as inputs. Initially, we replaced the MSSM block with a 1 x 1 convolutional block, then used the MSSM block alone, and finally employed the MSSM block along with the MCIF block. As shown in Table 6, the MSSM and MCIF blocks significantly improved the performance. The MSSM block enhanced the R^2^ by 0.4−0.9% and reduced the RMSE by 1.2−7.8% when predicting the seven soil properties. The MCIF block increased the R² by 0.7−2.6% and decreased the RMSE by 3.4−11.0%. These results indicate that the MSSM and MCIF blocks can improve the predictive performance of CNN, regardless of whether single-channel or multi-channel 2D images are used as input. This confirms the effectiveness of the MSSM and MCIF blocks. Our findings suggest that the proposed spatial attention mechanism enhances the feature extraction abilities of CNNs, leading to an improved soil property prediction performance.

**Figure 9:**
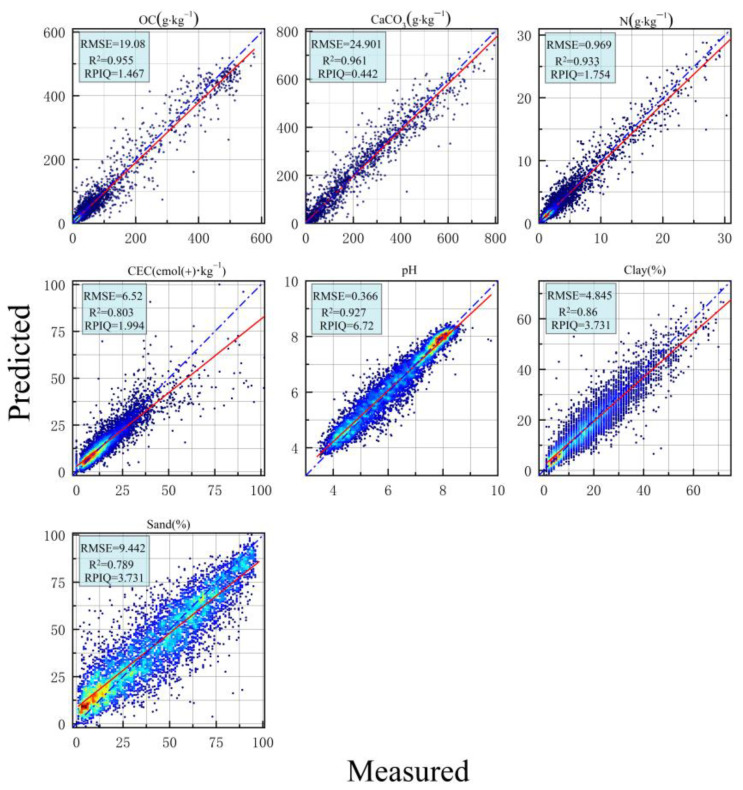
Scatter plot of CNNSANet model for measured and predicted values of seven soil properties.

Figure 9 presents scatter plots of the measured versus predicted values for the seven soil properties using the CNNSANet model, effectively illustrating their distribution. Among the predicted soil properties, CaCO_3_ and OC demonstrate the highest prediction accuracy (R^2^ > 0.95). The best models for predicting N and pH achieve R^2^ values of 0.935 and 0.93, respectively. However, the predictive performance for CEC and Clay is comparatively weaker, with R^2^ values of 0.803 and 0.86, respectively, while Sand shows the lowest R^2^ value of only 0.789.

### 3.3. Comparisons of Different Methods

**Figure 10:**
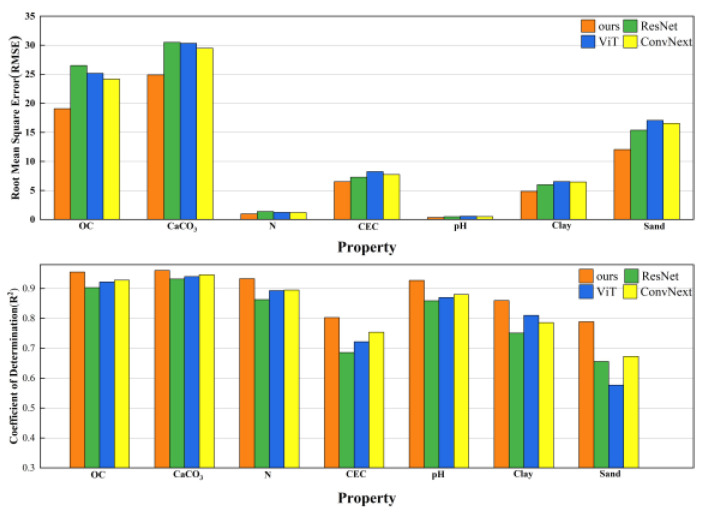
Results of the CNNSANet and other deep learning models for soil property prediction.

To demonstrate the superior performance of our model, we utilized the same optimal multi-channel 2D inputs for each soil property employed by other image processing models and conducted comparative analyses. We selected several representative algorithmic models: ResNet50, a deep convolutional network; Visual Transformer (ViT) [36], which combines natural language processing with image processing; and ConvNeXt, a next-generation convolutional neural network. Under consistent network hyperparameters, these models were trained to predict soil properties. The results of the soil property prediction performance (RMSE and R^2^) are presented in Figure 10. The results indicate that our model outperforms other models and can be effectively used for soil property prediction.

To further evaluate the predictive performance of our proposed modeling method on the soil attribute content, we compared the CNNSANet model with the two-dimensional convolutional neural network (2D-CNN) employed by Padarian et al. [18], the one-dimensional long short-term memory neural network (1D-LSTM) used by Singh and Kasana et al. [16], the two-dimensional Swin Transformer network (2D-Swin Transformer) utilized by Jin et al. [20], and the one-dimensional machine learning model (1D-PCR-Poly) proposed by Tavakoli et al. [37]. As shown in Table 7, the CNNSANet model significantly improves the prediction performance for most soil properties. Compared to the 2D-Swin Transformer, which also uses 2D transformation, our model reduces the RMSE for OC, N, CEC, pH, Clay, and Sand by 17.9%, 23.1%, 23.7%, 32.2%, 21.1%, and 21.3%, respectively. This improvement is attributed to the multi-channel 2D images we constructed, which enhance the input information. Additionally, our proposed convolutional neural network, featuring multi-scale spatial attention, offers stronger feature extraction capabilities, leading to better feature fitting and a higher prediction accuracy. It should be noted that some studies utilized both organic and mineral soils from the dataset [18,20,37], while others focused only on mineral soils [17,26]. Our approach considers organic and mineral soils as a single entity to enhance the model’s generalization performance.

## 4. Conclusions

This study proposes a CNN structure based on 2D multi-channel inputs and a multi-scale spatial attention mechanism. Firstly, we find that the combination of multi-channel inputs and 2D spectral inputs effectively improves the prediction accuracy of various soil properties. We investigate the impact of different channel numbers of 2D inputs for seven properties on the prediction results for each property. Additionally, our proposed convolutional neural network model with spatial attention mechanism, CNNSANet, can better capture the spatial positional correlation information of 2D spectral images, enhancing the feature extraction capability of the convolutional neural network, thereby improving the prediction of soil properties. For the large-scale LUCAS dataset, the CNNSANet model improves the prediction accuracy and outperforms current methods. Unlike laboratory data, VNIR spectra collected in the field are influenced by multiple environmental factors such as the weather, light intensity, and humidity. These factors can introduce higher data variability, thus complicating soil property prediction. Based on the favorable results obtained in this study, we will evaluate our model using more challenging field-collected soil VNIR spectra in future research.

## Figures and Tables

**Table 1:** 1D and 2D-VGG16 network architecture.

1D_Vgg16	2D_Vgg16
Input (1 × 4200)	Input (C × 64 × 64)
Conv1d 3-64	Conv2D 3 × 3-64
Conv1d 3-64	Conv2D 3 × 3-64
Maxpooling 2	Maxpooling 2 × 2
Conv1d 3-128	Conv2D 3 × 3-128
Conv1d 3-128	Conv2D 3 × 3-128
Maxpooling 2	Maxpooling 2 × 2
Conv1d 3-256	Conv2D 3 × 3-256
Conv1d 3-256	Conv2D 3 × 3-256
Conv1d 3-256	Conv2D 3 × 3-256
Maxpooling 2	Maxpooling 2 × 2
Conv1d 3-512	Conv2D 3 × 3-512
Conv1d 3-512	Conv2D 3 × 3-512
Conv1d 3-512	Conv2D 3 × 3-512
Maxpooling 2	Maxpooling 2 × 2
Conv1d 3-512	Conv2D 3 × 3-512
Conv1d 3-512	Conv2D 3 × 3-512
Conv1d 3-512	Conv2D 3 × 3-512
Maxpooling 2	Maxpooling 2 × 2
FC Dense	FC Dense

Note: C: The number of channels in two-dimensional input data; Conv1d 3-64: 1D convolutional layer with a kernel size of 3, outputting 64 channels; Conv2D 3 ×3-64: 2D convolutional layer with a kernel size of 3 × 3, outputting 64 channels; Maxpooling 2 × 2: 2D max pooling with a pool size of 2 × 2; FC Dense: fully connected layer.

**Table 2:** The number of permutations and combinations of different preprocessing methods after two-dimensional transformation.

CN	PCN	Abbreviation	CN	PCN	Abbreviation
1	8	NCC_1_	5	56	NCC_5_
2	28	NCC_2_	6	28	NCC_6_
3	56	NCC_3_	7	8	NCC_7_
4	70	NCC_4_	8	1	NCC_8_

Note: CN indicates the number of channels considered; PCN indicates the number of outcomes from permutation and combination; NCC indicates the number of combined channels.

**Table 3:** Information statistics of seven soil properties for training and testing sets.

Soil Properties	Valid Samples	Training								Testing							
		Samples	Min	Q1	Q2	Q3	Max	Mean	Standard Deviation	Samples	Min	Q1	Q2	Q3	Max	Mean	Standard Deviation
OC (g·kg^−1^)	19,036	13,325	0	12.7	20.8	39.3	586.8	50.17	91.85	5710	0	12.7	20.6	40.7	577	49.62	90.03
CaCO_3_ (g·kg^−1^)	19,036	13,325	0	0	1	12	944	51.31	124.75	5710	0	0	1	11	909	52.29	126.63
N (g·kg^−1^)	19,036	13,325	0	1.2	1.7	1.9	38.6	2.92	3.76	5710	0	1.2	1.7	2.9	34.2	2.93	3.74
pH	19,036	13,325	3.21	5.02	6.2	7.47	10.08	6.2	1.35	5710	3.41	5.01	6.22	7.47	9.75	6.2	1.35
CEC (cmol(+)·kg^−1^)	19,036	13,325	0	7	12.4	20.4	234	15.77	14.39	5710	0	7.1	12.3	20.1	227.7	15.7	14.7
Clay/%	17,939	12,557	1	8	17	27	79	18.84	13.02	5382	1	8	17	26	79	18.99	12.95
Sand/%	17,939	12,557	1	20	42	64	98	42.89	26.03	5382	1	19	42	64	98	42.81	26.24

**Table 4:** Test set results of seven soil properties (OC, N, CEC, pH, CaCO_3_) for single-channel 2D input constructed using different preprocessing methods based on the Vgg16 network model.

Preprocessing Algorithm	OC		CaCO_3_		N		CEC		pH		Clay		Sand	
	R^2^	RMSE	R^2^	RMSE	R^2^	RMSE	R^2^	RMSE	R^2^	RMSE	R^2^	RMSE	R^2^	RMSE
Absorbances	0.928	24.202	0.933	32.72	0.887	1.259	0.724	7.72	0.87	0.487	0.801	5.785	0.687	14.669
SNV + Detrend	0.925	24.62	0.935	32.16	0.89	1.242	0.732	7.613	0.863	0.501	0.776	6.128	0.651	15.506
sg0 + SNV	0.925	24.705	0.935	32.329	0.887	1.259	0.718	7.813	0.873	0.481	0.802	5.767	0.685	14.73
sg1 + SNV	0.922	25.13	0.937	31.902	0.88	1.296	0.713	7.881	0.887	0.454	0.709	6.989	0.667	15.151
sg2 + SNV	0.926	24.54	0.938	31.422	0.881	1.29	0.717	7.824	0.885	0.458	0.701	7.0787	0.647	15.59
sg0 + MSC	0.924	24.75	0.936	32.129	0.892	1.231	0.725	7.708	0.883	0.462	0.807	5.693	0.685	14.728
sg1 + MSC	0.922	25.119	0.935	32.187	0.874	1.331	0.709	7.927	0.885	0.458	0.71	6.979	0.662	15.248
sg2 + MSC	0.925	24.66	0.938	31.538	0.876	1.32	0.697	8.09	0.877	0.473	0.683	7.292	0.655	15.411

**Table 5:** The highest accuracy and multi-channel combination method for different multi-channel numbers based on different properties.

Soil Property	CN	Preprocessing Algorithm Combination	R^2^	RMSE
OC	3	SG0 + SNV, SG1 + MSC, SG2 + MSC	0.937	22.627
CaCO_3_	7	SG0 + MSC, SG0 + SNV, SG1 + SNV, SNV + DT, SG1 + SNV, SG1 + MSC, SG2 + MSC	0.948	28.941
N	7	SG0 + SNV, SG0 + MSC, SG1 + SNV, SG2 + SNV, SNV + DT, SG1 + MSC, SG2 + MSC	0.908	1.133
CEC	6	Absorbances, SNV + DT, SG1 + SNV, SG2 + SNV, SG1 + MSC, SG2 + MSC	0.782	6.863
pH	8	Absorbances, SG0 + MSC, SG0 + SNV, SG1 + SNV, SG2 + SNV, SNV + DT, SG1 + MSC, SG2 + MSC	0.896	0.436
Clay	5	Absorbances, SG1 + SNV, SG2 + SNV, SG1 + MSC, SG2 + MSC	0.812	5.609
Sand	6	Absorbances, SG1 + SNV, SG2 + SNV, SG1 + MSC, SG2 + MSC, SG0 + SNV	0.717	14.086

**Table 6:** The results of the ablation experiments on the MSSM block and MCIF block, using single-channel 2D images constructed from raw spectra and multi-channel 2D images constructed with the optimal preprocessing method for each soil property.

Soil	1 × 1 Conv2D (SC)		MSSM Block (SC)		MSSM Block + MCIF Block (SC)		1 x 1 Conv2D (MC)		MSSM Block (MC)		MSSM Block + MCIF Block (MC)	
Property	RMSE	R^2^	RMSE	R^2^	RMSE	R^2^	RMSE	R^2^	RMSE	R^2^	RMSE	R^2^
OC	23.965	0.929	22.07	0.94	20.776	0.947	22.13	0.94	21.34	0.944	19.08	0.955
CaCO_3_	31.321	0.939	29.133	0.947	27.428	0.953	28.99	0.948	26.73	0.955	24.9	0.961
N	1.24	0.89	1.13	0.909	1.065	0.919	1.16	0.904	1.09	0.915	0.97	0.933
CEC	7.36	0.749	7.183	0.761	6.931	0.778	6.9	0.78	6.75	0.789	6.52	0.803
pH	0.469	0.879	0.412	0.907	0.39	0.917	0.4	0.912	0.39	0.917	0.37	0.927
Clay	5.849	0.796	5.35	0.829	5.14	0.846	5.31	0.83	5.22	0.838	4.85	0.86
sand	15.268	0.661	13.883	0.72	13.21	0.749	13.26	0.745	13.1	0.751	12.06	0.789

Note: SC indicates the input of single-channel 2D images based on raw spectra, whereas MC indicates the input of multi-channel 2D images constructed with the optimal preprocessing methods for each attribute.

**Table 7:** The comparison between the proposed CNNSANet model in this paper and other methods from previous studies.

Model	Assessment Indicators	OC	CaCO_3_	N	CEC	pH	Clay	Sand
CNNSANet (this study)	RMSE	19.083	24.901	0.969	6.52	0.366	4.845	12.062
	R^2^	0.955	0.961	0.933	0.803	0.927	0.86	0.789
	RPIQ	1.467	0.442	1.754	1.994	6.72	3.715	3.731
2D-CNN [18]	RSME	32.14	NA	1.54	8.58	0.5	7.55	18.15
	R^2^	0.88	NA	0.83	0.66	0.87	0.7	0.53
1D-LSTM [16]	RSME	23.25	NA	1.15	6.75	0.42	NA	NA
	R^2^	0.94	NA	0.91	0.77	0.9	NA	NA
2D-Swin Transformer [20]	RMSE	23.25	NA	1.26	8.55	0.54	6.14	15.33
	R^2^	0.95	NA	0.94	0.79	0.9	0.84	0.74
	RPIQ	1.32	NA	1.27	1.25	5.2	2.77	2.74
1D-PCR-poly [37]	RMSE	21.33	25.71	1.11	6.89	NA	5.41	13.41
	R^2^	0.95	0.96	0.92	0.8	NA	0.82	0.73
	RPIQ	1.28	0.43	1.54	1.88	NA	3.33	3.28

Note: NA, not available.

## Data Availability

The authors do not have permission to share data.
